# Comorbid Substance Use and Mental Health Disorders: Prior Treatment/Admission as a Predictor of Criminal Arrest Among American Youths

**DOI:** 10.7759/cureus.21551

**Published:** 2022-01-24

**Authors:** Stanley Nkemjika, Eniola Olatunji, Connie Olwit, Oluwole Jegede, Colvette Brown, Tolu Olupona, Ike S Okosun

**Affiliations:** 1 Population Health Sciences, School of Public Health, Georgia State University, Atlanta, USA; 2 Psychiatry and Behavioral Sciences, Interfaith Medical Center, Brooklyn, USA; 3 Pharmacoepidemiology and Pharmacoeconomics, Brigham and Women's Hospital, Boston, USA; 4 Health Promotion and Behavior/Mental Health, School of Public Health, Georgia State University, Atlanta, USA; 5 Psychiatry, Yale School of Medicine, New Haven, USA; 6 Violence Prevention, Centers for Disease Control and Prevention, Atlanta, USA

**Keywords:** criminal arrest, treatment, mental disorder, substance use disorder, comorbid

## Abstract

Background: There is a dearth of literature with regards to substance use disorder (SUD) treatment outcomes and criminal arrest relationships.

Aim: We aimed to examine the association between criminal arrest within a month prior to SUD treatment admissions among 12- to 24-year-old Americans and the role of recurrent or prior SUD treatment.

Methods: The 2017 United States Substance Abuse and Mental Health Services Administration (SAMHSA) Treatment Episode Data Set - Admissions (TEDS-A; N = 333,322) was used for this analysis. Prevalence odds ratios from the multivariate logistic regression analyses were used to determine associations between recurrent or prior SUD treatment and criminal arrest one month before admission, adjusting for selected independent variables.

Results: Prior history of SUD treatment remained associated with past criminal arrest (adjusted OR = 0.972; 95% CI: 0.954-0.991; P = 0.004) after adjusting for gender, marital status, employment status, and source of income. Comorbid SUD-mental disorder was associated with past criminal arrest (adjusted OR = 1.046; 95% CI: 1.010-1.083; P = 0.012) after adjusting for gender, marital status, employment status, education, and source of income.

Conclusion: Our study shows that there is a protective association between history of previous substance treatment re-admissions and its relationship with criminal arrest one month before admission.

## Introduction

The joint occurrence of substance use disorder (SUD) and mental disorder (MD) is well documented in the literature [1,2] and is usually referred to as co-occurring disorder or formally called dual diagnosis. Comorbid SUD is a unique disorder being diagnosed with SUD as well as other mental health disorders at the same time. Analysis by Han et al. reported a prevalence rate of comorbid conditions to be about 3% (7.7 million) among US adults [3]. Despite documented evidence that SUD is a treatable condition of the brain [4,5], and that MD is a manageable condition [6], most individuals do not receive treatments [7,8]. Indeed, Compton et al.’s study indicated that half of the subjects suffering from both SUD and MD did not receive any form of treatment in the period between 2008 and 2014 [9]. Though the reasons for the lack of treatment are not clear, evidence in the literature has reported clients’ differences in perceptions of need, the role of stigma, financial inequalities, availability of human resources, and medical services as predictors [10]. Additionally, lack of treatment may also be associated with a lack of national policies guiding the treatment programs in subjects with comorbid conditions [11]. Other notable findings reported that barriers to treatment stem from lack of readiness among clients to stop using substances and deniability of the insured SUD clients [12]. Thus, not making treatment a priority.

It is noteworthy that SUD initiation time is more prevalent among youth and teenage years [13], as the burden of evidence in literature also suggests that SUD is a risk factor for aggressive behavior among youths [14]. Physiologically, the conceptual link between violence and SUD has been described as the pharmacological alterations of substances on dopamine homeostasis; thus, affecting an individual’s baseline functioning [14]. Based on this relationship, supportive evidence suggests that many SUD treatment programs often do not accept clients with past violence records or with high tendencies for violence [15]. Thus, suggesting that prior criminal records are an important reason for the lack of treatment in subjects with SUD and MD comorbidity [16]. Other studies have indicated that patients with more serious arrest histories may have a poorer functioning ability due to alcohol and other substance abuse intoxication [17]. Findings from the Swedish nationally representative sample indicated that crime offenders had a more significant reduction in risk of committing new crimes after receiving SUD treatment (HR = 0.325) than a reference category of those not receiving any SUD treatment [18]. To date, there is still paucity in the literature to support or refute this index study, especially in the American population. It is noteworthy that criminal arrest sometimes results from illegal drug-seeking behaviors [19] and disrupted behavior cues from brain changes triggered by repeated substance use [20]. Hence, studying the association between substance use and criminal arrest may help stakeholders strengthen substance use treatment programs for inmates or juveniles in the criminal system. This would provide continuity in treatment, hence reducing SUD and related comorbidities.

Substance use-related violence risk factors include younger age, being male, low income, history of violence and juvenile detention, previous physical abuse by parents/guardians, neighborhood influence, lack of social support, and unemployment [21]. Other seldomly reported substance use-related violence risk factors as evidenced in literature for adults on criminal arrest probation are drug-related violations and insufficient SUD-related treatments [22]. Similarly, findings from Claro et al. indicated that patients with co-occurring disorders were more likely to report SUD treatment problems, lack of medication adherence, and poor treatment outcomes [23].

Despite the recognized importance of the treatment of SUD and MD comorbidity in curbing the prevalence of crime and arrest, to the best of our knowledge, there is no evidence in the literature examining the association between recurrent or prior SUD treatment and criminal arrest. Secondly, there are limited updates with regards to biological sex differences. Hence, this study aims to examine the relationship between criminal arrest within a month prior to SUD treatment admissions among 12- to 24-year-old Americans and the role of recurrent or prior SUD treatment. Also, we aim to look at the biological sex prevalence of the Treatment Episode Data Set - Admissions (TEDS-A). We hypothesize that teenage subjects with multiple prior SUD treatments or admissions will be more likely to be arrested within a month than subjects with no prior SUD treatments or admissions. We also posit that the odds of criminal arrest in teenagers with comorbid disorders will be compared to teenagers without comorbid conditions.

## Materials and methods

Sample

Data for this study were drawn from the public use data files of TEDS-A for the year 2017 (n = 2,005,395) of participants between the age of 12 years and above. TEDS-A is a census of all admissions to treatment facilities reported to the Substance Abuse and Mental Health Services Administration (SAMHSA) by state substance abuse agencies. TEDS-A collects data on admissions to substance abuse treatment facilities across the United States that can be used to examine differences in the primary substance of abuse among males and females by age and ethnicity. TEDS-A can also be used to estimate the prevalence of SUD and MD comorbidity, the history of previous admissions, and re-admissions for SUD treatment episodes. In our analyses, we restricted the sample to participants 12-24 years of age (n = 333,322), which caters to the age range with the most burden of substance use initiation and burden.

Ethics

Since we utilized a secondary dataset that is de-identified and publicly available on the SAMHSA website, institutional review board approval was not required.

Measures

Dependent Variable: Previous Arrest (ARREST)

In TEDS-A, participants were asked to report the “Number of arrests in the 30 days prior to admission.” The response includes “None,” “Once,” or “Once or twice.” For this analysis, “ARREST” was defined as answering “Once” or “Once or twice.”

Independent Variables

The main independent variable for this study is the number of previous substance use treatment episodes (NOPRIOR). In TEDS-A, participants were asked to report the “Number of previous substance use treatment episodes they have received in any drug or alcohol treatment programs.” The responses comprised of “No prior treatment episodes,” “One prior treatment episode,” “Two prior treatment episodes,” “Three prior treatment episodes,” “Four prior treatment episodes,” “Five or more prior treatment episodes.” For this analysis, the number of prior substance use treatment episodes is three or more versus two or less.

SUD and MD comorbidity (PSYPROB): In this study, we defined PSYPROB based on participants' psychiatric history in addition to the history of alcohol or drug use. Participants responded as “Yes” or “No.”

Socio-demographics: The social demographic variables that were included in this study are age in categories (age at admission), gender, race/ethnicity, marital status, education, source of income, substance use while on admissions, living arrangement, and region of the country.

Data analysis

All analyses for this study were conducted using SAS 9.4 statistical software (SAS Institute Inc., Cary, NC). Analyses included descriptive statistics of the population and frequency distributions of the dependent variable; each independent variable and all covariates were ascertained. After assessing the distribution of each measure, we conducted bivariate chi-square (χ2) analyses to determine the presence of a relationship between each independent variable and covariate with the previous arrest within 30 days prior to admission. We also compared basic demographics and independent variables across gender using the chi-square test to determine gender differences. Bivariate logistic regressions were conducted to determine factors that are associated with a criminal arrest. We also used the odds ratio (OR) from the multivariate logistic regression analysis to determine the treatment of SUD, comorbid condition, and prior criminal arrest, adjusting for gender, marital status, source of income, and employment status. All estimates for this analysis were based on a 95% confidence interval and a p-value < 0.05.

## Results

Characteristics of our study population

The basic characteristics of study populations are shown in Table 1. The majority (77.2%) of our participants were between 18 and 24 years old, and 63.34% were male. Of the participants, 39% had attained the General Educational Development test (12 years at school). Most (63.4%) of the participants were Whites, followed by Asians (15.78%) and Blacks (14.93%). Regarding the source of income, 21.79% had no source of income, and 33.67% had a source of income. It is interesting to note that among our sample, 15.82% of the participants continuously took alcohol as they were admitted, and 78.33% continued to use other substances of abuse while on treatment admission.

**Table 1:** Demographic distribution of teenagers (N = 333,322) on SUD treatment admissions in the United States of America for 2017. GED - General Educational Development.

Characteristics	Frequency	Percentage (%)
Age range (years)		
12-14	12,843	3.85
15-17	63,115	18.94
18-20	70,343	21.1
21-24	187,021	56.11
Gender		
Male	211,110	63.34
Female	121,896	36.57
Missing	316	0.09
Marital status		
Never married	224,699	67.41
Presently married	9,157	2.75
Separated/divorced	4,427	1.33
Missing	95,039	28.51
Ethnicity		
Alaskan Native	7,992	2.4
American Indian	3,547	1.06
Black	49,765	14.93
White	211,322	63.4
Asian	52,608	15.78
Missing	8,088	2.43
Years in school		
8 years or less	26,323	7.9
9-11 years	114,013	34.2
12 years (GED)	128,266	38.48
13-15 years	34,528	10.36
16 years or more	4,334	1.3
Missing	25,858	7.76
Primary income		
Wages/salary	58,993	17.7
Public assistance	9,488	2.85
Retirement/disability	2,366	0.71
Other	38,048	11.41
None	72,631	21.79
Missing	151,796	45.54
Living arrangement		
Homeless	26,581	7.97
Dependent living	103,592	31.08
Independent living	175,217	52.57
Missing	27,932	8.38
Continuous substance use at admission		
None	5,006	1.5
Alcohol	52,712	15.82
Other substances	261,095	78.33
Missing	14,509	4.35
Previous substance use treatment admission		
None	144,796	43.44
Once	70,207	21.06
Twice	30,029	9.01
Three times	15,811	4.74
Four times	8,438	2.53
Five or more times	19,589	5.88
Unknown	44,452	13.34
Comorbid mental disorder and substance use disorder		
No	171,089	51.33
Yes	93,538	28.06
Missing	68,695	20.61
Prior criminal arrests		
None	243,442	73.04
Once	24,793	7.44
Twice or more	3,799	1.14
Missing	61,288	18.38

The findings show that 36.15% of our study participants had comorbid conditions, while 49.88% had a treatment history of SUD. Among the Blacks, there were more males than females with substance use (17% vs. 12%) while among the Whites, it was contrary with 69% of females and 63% of males with substance use. Relapse of SUD was similar across gender (49.6% vs. 50.4%); however, female participants had more ≥2 relapses than males (27% vs. 25%) while the comorbid condition was higher among females compared to males (41.7% vs. 31.8%). It is interesting to note that more males had SUD than females at the age of 15-17 years (21% vs. 16%). On the contrary, at the age of 21-24 years, more females were admitted for SUD than males (59% vs. 54%). See Table 2 for more details.

**Table 2:** Age, race, and independent variables by gender distribution for substance use disorder treatment admissions (N = 333,322). * Recurrent Rx - previous substance use treatment admission; Mental & SUD - comorbid mental disorder and substance use disorder.

Characteristics	Male (N = 211,110)	Female (N = 121,896)	Total	P-value
					Age in years (N = 333,006)				<0.001
12-14	7,799 (3.69)	5,021 (4.12)	12,820 (3.85)						
15-17	43,315 (20.52)	19,732 (16.19)	63,047 (18.93)						
18-20	45,417 (21.51)	24,852 (20.39)	70,269 (21.10)						
21-24	114,579 (54.27)	72,291 (59.31)	186,870 (56.12)						
Race (N = 324,972)				<0.001
Indian/Alaskan Native	4,309 (2.09)	3,675 (3.09)	7,984 (2.46)	
Asian	2,377 (1.15)	1,170 (0.98)	3,547 (1.09)	
Black	35,305 (17.14)	14,442 (12.13)	49,747 (15.31)	
White	129,110 (62.70)	82,028 (68.90)	211,138 (64.97)	
Others	34,823 (16.91)	17,733 (14.90)	52,556 (16.17)	
Recurrent RX (N = 288,725)				<0.001
None	93,092 (50.41)	51,618 (49.60)	144,710 (50.12)	
Once	45,606 (24.71)	24,572 (23.61)	70,178 (24.31)	
Twice or more	45,957 (24.89)	27,880 (26.79)	73,837 (25.57)	
Mental & SUD (N = 264,473)				<0.001
No	115,198 (68.22)	53,666 (58.35)	168,864 (63.85)	
Yes	55,784 (31.78)	39,825 (41.65)	95,609 (36.15)	

Prevalence of criminal arrests within a month based on our demographics

Generally, 10.5% of our study sample was arrested in the previous 30 days (Table 1), increasing with age. More males with SUD than those with no SUD had more arrest frequency than females (68% vs. 64% for males and 32% vs. 36% for females, respectively). Table 3 shows the sociodemographic attributes of the participants by criminal arrest status. Among participants of Alaskan origin, arrests were more (4% vs. 2%), while among the Blacks, there were fewer arrests (15% vs. 17%). Furthermore, those dependent on housing were less likely to be arrested than those who lived independently. There were similar arrests for those who had been in school for 9-11 years and those who had spent 12 years at school. Similarly, the arrests were fewer among those who had a salary/wage.

**Table 3:** Prevalence of socio-demographics by past criminal arrest status across all independent variables (N = 272,034). * Admission SUD - the continuous use of substance while on treatment admission; Previous Rx - prior SUD treatment admissions; Mental & SUD - comorbid mental disorder and substance use disorder; GED - General Educational Development.

Characteristics	% No arrest (N = 243,442)	% Arrest (N = 28,592)	Total	P-value
Age range in years (N = 272,034)				<0.0001
12-14	3.29	3.01	8,874	
15-17	17.38	21.4	48,430	
18-20	21.66	22.29	59,095	
21-24	57.67	53.3	155,635	
Gender (N = 271,933)				
Male	63.71	67.78	174,413	<0.0001
Female	36.29	32.22	97,520	
Marital status (N = 228,076)				<0.0001
Never married	94.48	95.17	215,654	
Presently married	3.65	3.16	8,214	
Separated/divorced	1.86	1.68	4,208	
Ethnicity (N = 266,685)				<0.0001
Alaskan Native	2.36	3.65	6,644	
American Indian	0.96	0.83	2,519	
Black	16.96	14.5	44,536	
White	66.66	68.03	178,164	
Asian	13.06	13	34,822	
Years spent at school (N = 265,821)				<0.0001
8 years or less	8.14	8.23	21,654	
9-11 years	35.9	39.92	96,551	
12 years (GED)	42.68	39.9	112,667	
13-15 years	11.77	10.74	31,000	
16 years or more	1.52	1.21	3,949	
Employment status (N = 264,653)				<0.0001
Not in the labor force	38.92	41.91	104,659	
Unemployed	34.59	33.42	91,562	
Part-time	10.22	9.6	26,399	
Full-time	16.27	15.07	42,033	
Primary income (N = 174,634)				<0.0001
Wages/salary	33.46	28.19	57,418	
Public assistance	5.39	4.04	9,153	
Retirement/disability	1.36	1.21	2,344	
Other	20.59	20.03	35,854	
None	39.2	46.53	69,865	
Housing (N = 264,960)				<0.0001
Homeless	7.64	8.85	20,581	
Dependent living	31.36	37.61	84,830	
Independent living	61.01	53.54	159,549	
Admission SUD (N = 270,678)				<0.0001
None	1.66	0.7	4,225	
Alcohol	17.16	17.4	46,520	
Other substances	81.18	81.9	219,933	
Previous Rx (N = 257,854)				<0.0001
None	49.28	48.33	126,818	
Once	24.47	24.62	63,136	
Twice	26.25	27.06	67,900	
Mental & SUD (N = 233,432)				<0.0001
No	61.45	59.41	142,966	
Yes	38.55	40.59	90,466	

Association of variables with one-month criminal arrest among US young adults

Table 4 shows the logistic regression analysis result that examined the relationship between past crime arrest and independent variables that include sociodemographic characteristics. On bivariate analysis, age group 15-17 years and 18-20 years showed protective association with criminal arrest with crude odds ratio (cOR) of 0.743 (95% CI: 0.689-0.801; P < 0.0001) and 0.889 (95% CI: 0.825-0.958; P = 0.002), respectively. Females were more likely to be arrested than males with cOR of 1.198 (95% CI: 1.167-1.230; P < 0.0001). Similarly, being married or separated were associated with crime arrest with cOR of 1.165 (95% CI: 1.080-1.258; P < 0.0001) and 1.120 (95% CI: 1.009-1.242; P = 0.033), respectively. Continuous substance and alcohol use during treatment admission were also associated with previous criminal arrest with cOR of 0.412 (95% CI: 0.357-0.477; P < 0.0001) and 0.414 (95% CI: 0.359-0.478; P < 0.0001), respectively.

**Table 4:** Association between variables and previous criminal arrests among young adults in the United States. * Reference level: age: 12-14; gender: male; marital status: never married; education: eight years or less; employment status: "not in the labor force"; substance use on admission: none. GED - General Educational Development.

Characteristics	Crude odds ratio	95% CI
Age in years		
15-17	0.743	0.689-0.801
18-20	0.889	0.825-0.958
21-24	0.99	0.921-1.064
Gender		
Female	1.198	1.167-1.230
Marital status		
Married	1.165	1.080-1.258
Separated	1.12	1.009-1.242
Education		
9-11 years	0.91	0.868-0.954
12 years (GED)	1.082	1.032-1.135
13-15 years	1.108	1.047-1.174
16 years or more	1.274	1.130-1.435
Employment status		
Unemployed	1.151	1.119-1.183
Part-time	1.494	1.425-1.566
Full-time	1.482	1.425-1.541
Substance use on admission		
Alcohol	0.412	0.357-0.477
Others	0.414	0.359-0.478

Equally, prior history of SUD treatment and comorbid condition was associated with the previous history of criminal arrest as the initial showed a protective relationship of cOR = 0.975 (95% CI: 0.961-0.990; P = 0.001), and the latter showed a harmful relationship of cOR = 1.089 (95% CI: 1.060-1.120; P < 0.0001), respectively. Using multivariate logistic analysis, prior history of SUD treatment remained statistically significant with an adjusted OR = 0.972 (95% CI: 0.954-0.991; P = 0.004) after adjusting for gender, marital status, employment status, and source of income. A similar trend was noted for comorbid SUD and MD as the association with past one-month crime arrest remained statistically significant (OR = 1.046; 95% CI: 1.010-1.083; P = 0.012) after adjusting for gender, marital status, employment status, education, and source of income. See Table 5 for details.

**Table 5:** Association between prior criminal arrest with previous treatment of SUD and comorbid mental disorder and SUD. * OR - odds ratio; Previous Rx - prior SUD treatment admissions; Mental & SUD - comorbid mental disorder and substance use disorder. + Adjusted for gender, marital status, employment, age, educational level, and source of income. ++ Adjusted for gender, marital status, source of income, and employment status.

Characteristics	Crude OR (95% CI)	P-value	Adjusted OR (95% Cl)	P-value
Previous Rx				
No	Ref		Ref	
Yes	0.975 (0.961-0.990)	0.001	0.972 (0.954-0.991)+	0.004
Mental & SUD				
No	Ref		Ref	
Yes	1.089 (1.060-1.120)	<0.0001	1.046 (1.010-1.083)++	0.012

## Discussion

Our study showed a negative relationship between substance use treatment history and previous one-month criminal arrest. The odds of arrest 30 days before admission of patients with one or more prior SUD treatment were 0.972 times the odds of arrest for patients with no prior SUD treatment history. This finding differs from our proposed research hypothesis. Hence, patients with previous treatment were less likely to be arrested 30 days before their present treatment admission. One possible explanation for this finding is that when a person has ever been treated, they are more aware of the initial behavioral and clinical changes associated with previous SUD episodes [24]. Therefore, they could seek help as soon as they could. Another possible explanation could be that they have been treated before, and they experienced the positive effects of treatment. Thus, they can seek help in a proactive manner, which will aid the evasion of violent acts that may lead to crime.

Our finding is supported by the results from Henggeler et al.'s (2002) study conducted among 80 SUD juvenile offenders [25]. They found that multisystemic therapy (MST) and community services reduced aggressive criminal activity though one was better than the other [25]. Another possible explanation could be the concept of “hitting bottom.” Chen (2018) refers to “hitting bottom” as a state where a person with SUD experiences a loss of personal and social resources [26]. This concept is referred to as a turning point for clients who want to reverse their SUD situation by seeking care, which could explain fewer criminal arrests attributable to previous treatment. Therefore, SUD treatment facilities and resources should be made available and expanded across countries. Additionally, during the implementation of such programs, existing literature should be used to guide practice. For example, our study found that females are more likely to seek treatment more than once than males and several hypotheses have explained biological sex differences in terms of hormonal and genetic makeup [27,28]. Therefore, males need more motivation to come back for treatment in case of relapse. There was also a biological sex difference in races regarding SUD, and this must be considered to target those who are more at risk for specific races. For example, we found that among the blacks, there were more males than the female who were using substances of abuse (17% vs. 12%), which was a contrast to Whites that had a higher prevalence among females than males.

Our study found a positive association between SUD and MD comorbidity and arrest history 30 days before admission. The odds of experiencing arrest 30 days prior to admission among those with SUD and MD comorbidity were 1.046 times the odds of those without SUD and MD comorbidity. Thus, suggesting that SUD-MD comorbidity increases chances of arrest 30 days prior to admission by 5%. This finding corroborates some of the literature studies regarding the increased likelihood of comorbid disorder patients being jailed or detained [29]. For example, Walter (2018) reported that youth who had a crime and SUD history had significantly higher scores on psychiatric symptoms such as neuroticism, grandiosity, unemotionality, impulsivity, and moral disengagement [30]. The presence of mental disorders may compromise the individuals' intellectual ability and decision-making; hence, increasing rates of being involved in criminal behaviors [30]. Therefore, screening of other mental illnesses should be emphasized during the treatment of SUD, and treatment should be offered. It is also crucial to screen for mental illness among youth history of crime so that treatment is provided as soon as possible to change the narratives.

The results of this study should be viewed in the context of its limitations. We could not ascertain details on violence because of the nature of our study design and the use of a secondary dataset, as this would have been informative in relation to the race of the clients. Though we were able to obtain a response for prior treatment episodes, we cannot ascertain the mental state of the participants at the time of collection of data or if the response was obtained from a database. The TEDS-A datasets are derived from patients who reported to government facilities or designated facilities, so it does not capture patients who report to non-registered facilities, those who live in shelters, or those who were managed at home. Additionally, some of the question constructs of the questionnaire may underestimate the relationship. For example, the “criminal arrest” variable according to TEDS-A is limited to the past 30 days and not beyond. This study also did not highlight causality and might not be generalizable due to the cross-sectional design. Our study possesses some merits as the database is generalizable since information is coming from all the states in the United States.

Irrespective of the limitations, this study is the first to provide insight into the relationship between SUD treatment and prior arrest on American youths using a nationally representative study. The most important merit is that this is the first study to look at the relationship between prior treatment for SUD and history of criminal arrest or violence. Secondly, it also highlights the biological sex disparity about SUD-related recurrent treatment prevalence rates. Thirdly, the collected data are not a sample, rather these data are holistic population information on those being treated for substance use disorder.

## Conclusions

Conclusively, this study offers important findings with implications about the history of previous substance use treatment re-admissions and its relationship with criminal arrest within one month. It also found an association between comorbid conditions and the prevalence of one-month unlawful arrest. It also proffers information on gender disparity for the re-admission rates of SUD treatment services. Our results are by no means causal but provide an insight into a trend or behavioral pattern. Hence, it is vital to consider this population with regard to policy change and management modality. Finally, we recommend that future surveys should consider more specific types of comorbid disorders present, for example, SUD with major depressive disorder (MDD) vs. SUD with anxiety vs. SUD with borderline personality disorder (BPD), etc.
